# American Scientific Association

**Published:** 1851-07

**Authors:** 


### AMERICAN SCIENTIFIC ASSOCIATION.

We had the pleasure of being present at the late meeting of the American Association for the Advancement of Science. The meeting took place at Cincinnati on the 5th of May and continued five days. We were particularly struck with the zeal with which the members prosecuted the business of the Association. Our impression is, that we have never attended the meeting of a body more in earnest, or more interested in the objects before it. The aim of the Association is the *advancement*, not the diffusion, of science, and consequently nothing is expected to be presented but what is an actual contribution to the existing stock of knowledge. Many new facts were brought out at the meeting in Cincinnati, the result of the researches of American naturalists, which will be embodied in the Transactions of the Association soon to be published.

The medical profession was fully and honorably represented at the meeting. Some of the brightest names in the Association belong to our profession, and the meeting owed a full share of its interest to their communications. Zoology, comparative anatomy, and physiology each came in for a share of illustration, and the facts with which they were enriched go to enlarge the boundaries of medical science. Medicine has a stake in every science which has for its objects the living world, and we may add, that it has its representatives in every body laboring for the extension of human knowledge. Now, as in all times past, physicians are among the foremost in the cultivation of science. Whatever new investigation is set on foot, physicians may be counted upon to take part in it. The latest born of the sisterhood of sciences—Ethnology and Geology—have found many of their most zealous and successful contributors in the members of our profession. Ethnology, is it not indeed the creation of medical men? But, we did not mean to grow vainglorious; we meant simply to advert to the fact that so many members of the Association are also members of our profession, as affording evidence that American physicians are not indifferent to the interests of general science. Y.

